# Dementia Caregiver Preparedness for Surrogate Decision-Making: Specificity and Confidence in Care Planning

**DOI:** 10.1093/geroni/igaf122.4285

**Published:** 2025-12-31

**Authors:** Rachel Bloom, Elizabeth Rojas, Emily LeRolland, Francesca Falzarano

**Affiliations:** Yale University, New York, New York, United States; University of Southern California, Los Angeles, California, United States; Fordham University, Bronx, New York, United States; University of Southern California, Los Angeles, California, United States

## Abstract

Dementia family caregivers commonly find themselves serving as medical surrogate decision-makers as the person with dementia nears their end of life. Engagement in advance care planning (ACP) conversations has been found to alleviate decision-making burden among surrogates, but these conversations rarely take place before the later stages of dementia. This study examines dementia caregivers’ (N = 34) surrogate decision-making preparedness, using a semi-structured interview to ask about care planning conversations with their family member and their role as potential surrogates. We developed and applied a novel content coding system to assess caregivers’ confidence and specificity in their planning approach and understanding of preferences. Survey data on self-efficacy, preparedness, mutuality, burden, and ACP engagement were also collected. We found that the presence of confidence was positively correlated with ACP engagement (r = .42, p = .01), while lack of confidence was negatively correlated with ACP engagement (r = -.46, p < .01). The presence of specificity was negatively correlated with self-efficacy in obtaining respite (r = -.38, p = .03), and lack of specificity was negatively correlated with burden (r = -.45, p < .01) and positively correlated with mutuality (r = .35, p < .05). The association between a lack of specificity and mutuality may reflect an assumption that specific planning is unnecessary when relationship quality is high. This work helps to elucidate the relationships between different aspects of surrogate decision-making for people with dementia and provides touchpoints for future decision-making preparedness research and interventions involving family caregivers.

